# Saponins from *Solanum nigrum* L. Fruit: Extraction Optimization, Structural Characterization, and Dual-Functional Efficacy

**DOI:** 10.3390/foods14132370

**Published:** 2025-07-03

**Authors:** Shuyuan Chen, Weiyun Guo, Tonghe Zhang, Jianfang Chen, Li Huang, Jihong Huang, Ruqiang Huang

**Affiliations:** 1 School of Life Science, South China Normal University, Guangzhou 510631, China; csycsy1031@163.com (S.C.); zhangtonghe0310@163.com (T.Z.); 13416409794@163.com (J.C.); 2 Food and Pharmacy College, Xuchang University, Xuchang 461000, China; gwy2002@126.com; 3 Collaborative Innovation Center of Functional Food by Green Manufacturing, Xuchang 461000, China; 4 Henan Institute of Food and Salt Industry Inspection Technology, Zhengzhou 450003, China; ssjyjwb@163.com

**Keywords:** Solanaceae, natural compound, anti-oxidation, bacteriostasis

## Abstract

*Solanum nigrum* L., a widely consumed Asian medicinal edible plant, is a promising source of bioactive saponins for functional food applications. This study optimized the extraction of saponins from *S. nigrum* fruits (8.59% total saponin yield), followed by isolation via column chromatography and structural elucidation using spectroscopic analyses (IR, NMR, and MS). Concurrently, the antioxidant properties and antibacterial activity of the purified substances were detected and analyzed. The three saponins (SNL1, SNL2, SNL3) were identified as γ_2_-Solamargine , Diosgenin, and β-Solanine. The  *n*-butanol -purified fraction demonstrated a remarkable capacity to scavenge DPPH, hydroxyl, and ABTS radicals (DPPH IC50 = 0.0096 mg/mL; hydroxyl radical IC50 = 0.8 mg/mL; ABTS IC50 = 0.061 μg/mL), indicating the inhibition of a multi-pathway oxidative chain reaction. Concurrently, the saponins exhibited selective antimicrobial efficacy against key foodborne pathogens, particularly Escherichia coli. To the best of our knowledge, this work provides the first empirical evidence of *S. nigrum* fruit saponins as dual-functional natural preservatives, synergistically suppressing lipid oxidation and microbial growth. These findings highlight their potential as safer, multi-mechanistic alternatives to synthetic additives, aligning with clean-label food industry demands.

## 1. Introduction

*Solanum nigrum* L., an erect annual herb of the Solanaceae family [1], produces spherical fruits that transition from green to black-violet during ripening, exhibiting palatable sweet-sour characteristics [2]. The primary toxic components in *Solanum nigrum* are glycoside alkaloids, such as solanine and solasodine. These compounds are toxic to humans at high concentrations and may cause symptoms such as nausea, vomiting, diarrhea, and headaches. In severe cases, they may even lead to breathing difficulties and heart problems. The concentration of glycoalkaloids in unripe *Solanum nigrum* fruits is higher, making them more toxic [2,3]. This species is widely distributed in temperate regions of Eurasia and the Americas [4,5]. In China, it is mainly found in Hebei, Sichuan, and the northeastern areas, with adaptability to marginal habitats such as field edges and slopes [3]. It is reported to be rich in bioactive components, which primarily include saponins, alkaloids, polysaccharides, and phenolic acids [6,7,8]. Consequently, the biological activities of *Solanum nigrum* are quite diverse, exhibiting not only antioxidant and anti-inflammatory properties [9] but also demonstrating potent anti-tumor [10,11], hypoglycemic [12,13], and lipid-lowering effects [14]. Given these properties, it holds potential as a source of natural preservatives for food systems, aligning with the growing demand for plant-derived bioactive compounds [15,16].

Current research on *S. nigrum* fruits predominantly focuses on polyphenols and alkaloids, while its saponins remain underexplored despite their structural diversity and functional versatility. Saponins are a kind of natural compound with diverse structures, consisting of two parts: saponins and glycosyl groups [17,18,19]. Steroidal aglycones typically feature a tetracyclic cyclopentanophenanthrene backbone, whereas triterpenoid aglycones exhibit pentacyclic structures [19,20,21,22]. The amphiphilic properties of saponins, that is, they are both hydrophilic and lipophilic, so that they can form bubbles in aqueous solutions, which is also the origin of the name “saponins” [23,24]. The molecular structure of saponins determines their biological activity and physicochemical properties, including anti-inflammatory, antioxidant, anti-tumor, immunomodulatory, and cardiovascular protection [17]. These properties of saponins provide wide application potential in the fields of medicine, food, cosmetics, and agriculture [25]. Notably, *S. nigrum* saponins exhibit unique steroidal configurations (spirostanol/furostanol types) [26,27], which may modulate membrane permeability in microorganisms.

Despite these prospects, some critical knowledge gaps persist: (1) absence of standardized purification protocols for *S. nigrum* fruit saponins, (2) insufficient structural validation of individual components, and (3) insufficient activity data. This study aims to bridge these gaps by employing a systematic approach that integrates process optimization, structural elucidation, and bioactivity analysis, providing foundational data for evaluating *S. nigrum* saponins as multifunctional food additives.

## 2. Materials and Methods

### 2.1. Materials and Reagents

*Solanum nigrum* fruits were purchased from Shenyang in the Liaoning Province of China (41.7961° N, 123.4168° E). Mature *Solanum nigrum* fruits with full fruit shape and no damage were selected, dried, pulverized, sieved with a 60 mesh sieve, and stored at room temperature.

*Staphylococcus aureus*, *Escherichia coli*, *Candida albicans*, *Shigella flexneri*, and *Pseudomonas aeruginosa* were purchased from the Guangdong Microbial Collection Center (Guangzhou, Guangdong, China).

Anhydrous ethanol, methanol, dichloromethane, chloroform, and *n*-butanol were purchased from Tianjin Damao Chemical Reagent Factory (Dongli District, Tianjin, China). DPPH was purchased from BJ Biotopped (Haidian District, Beijing, China). The ascorbic acid was purchased from Shanghai Aladdin Biochemical Technology Co., Ltd. Vanillin (Fengxian District, Shanghai, China) and ABTS were purchased from Shanghai Macklin Biochemical Technology Co., Ltd. (Fengxian District, Shanghai, China). Glacial acetic acid, perchloric acid and salicylic acid were purchased from Guangzhou Chemical Reagent Factory (Guangzhou, Guangdong, China). Plate count agar and nutrient broth medium were purchased from Guangdong Huankai Microbial Sci. & Tech. Co., Ltd. (Guangzhou, Guangdong, China). All other reagents were analytically pure.

The ginsenoside Re standard used in this experiment was produced by Shanghai Yuanye Biotechnology Co., Ltd. (Songjiang District, Shanghai, China), HPLC ≥ 98%. 

### 2.2. Determination of Total Saponin Content

The detection of total saponins was referred to [28] with some modifications.

Ginsenoside Re was employed as the standard product. A 1 mg/mL ginsenoside Re standard solution was prepared for utilization. The standard solutions were diluted with methanol to an appropriate concentration. An amount of 0.5 mL of standard solutions of various concentrations was dried in a 10 mL cuvette; then, 200 μL of 5% vanillin–glacial acetic acid solution was added, followed by 800 μL of perchloric acid, and the solution was shaken uniformly. Subsequently, it was placed in a 65 °C water bath for 15 min and immediately transferred to ice water for cooling for 3 min; 5 mL of glacial acetic acid was added and shaken well. Absorbance values were measured at 545 nm. The abscissa represents the concentration of the standard solution, and the ordinate represents the absorbance for drawing the standard curve.

Determination of the total saponin content of the sample: The sample solution was lyophilized and dissolved with a certain quantity of methanol. A total of 0.5 mL of the sample solution was weighed in a 10 mL cuvette and dried with methanol. The subsequent steps were the same as those mentioned above. The formula for calculating the total saponin yield is shown in Equation (1). The total saponin yield refers to the percentage of the total saponin mass obtained during the extraction process relative to the mass of the raw material used for extraction, and is used to measure the efficiency of extracting total saponins from the raw material.

(1) $$\mathrm{The}\;\mathrm{total}\;\mathrm{saponin}\;\mathrm{yield}\;(\%)=\frac{C\times V}{M}\times 100\%$$

where *C*  represented the saponin concentration, *V* represented the volume of the test liquid, and *M* represented the mass of the sample taken. 

### 2.3. Optimization of the Extraction Process of Crude Saponins 

A combination of single-factor and orthogonal test methods was used for optimization. The single-factor experiments were performed in sequence, as detailed in Table S1. The orthogonal experimental factor level settings are shown in Table S2. 

### 2.4. Isolation and Purification of Saponins

Concentrate the crude extract obtained under optimal conditions to obtain a crude saponin extract from *Solanum nigrum* fruit. Subsequently, extract with petroleum ether to remove lipid-soluble impurities, then extract with water-saturated n-butanol; concentrate to obtain an *n*-butanol phase extract and take an appropriate amount for vacuum drying for subsequent functional tests. We used the vacuum freeze dryer manufactured by Christ in Osterode , Germany , model Alpha1-8LD Plus. Finally, the extract diluted with methanol was precipitated using acetone–diethyl ether (1:1), yielding 83.4 g of precipitation (saponins). An appropriate amount was vacuum-dried for subsequent functional tests. 

Crude saponins underwent sequential fractionation commencing with petroleum ether-aqueous partitioning, followed by exhaustive extraction using water-saturated n-butanol. The resultant *n*-butanol phase was concentrated under reduced pressure, resolubilized in methanol, and precipitated via methanol–acetone-ether (7:3) with subsequent centrifugation (169,833× *g*, 10 min). Silica gel adsorption chromatography was employed for primary separation, with fraction pooling guided by thin-layer chromatography (TLC) monitoring. 

We screened the dichloromethane–methanol system as the eluent. When eluting at a constant ratio of 85:15, the collected fractions were analyzed by TLC, and fractions 1–19 were combined to form Fr. 1, while fractions 20–57 formed Fraction 2. Fraction 2 was concentrated and subjected to column chromatography again, eluting with chloroform–methanol (85:15), and the fractions were combined to obtain the key components: Fraction 2-2 (fractions 14–39) and Fraction 2-4 (fractions 60–64, SNL2). Fraction 2-2 was further purified by gradient elution (90:10 → 85:15), and the fractions 23–30 were combined and concentrated to obtain the target product Fraction 2-2-2 (SNL1). When eluting at an 80:20 ratio, the fractions were combined and analyzed to obtain Fr. 3 (fractions 1–15), Fraction 4 (fractions 16–54), and Fr. 5 (fractions 55–72). 

Among these, Fraction 4 was subjected to secondary purification to obtain three subcomponents, and its core component Fraction 4-2 (fractions 24–49) was subjected to gradient elution (85:15 → 80:20). After merging and concentrating fractions 29–34, Fraction 4-2-2 (SNL3) was successfully separated. The purity of all products was verified by thin-layer chromatography and liquid chromatography. 

### 2.5. Analysis and Characterization of Purified Substance

#### 2.5.1. Thin-Layer Chromatography

We placed a thin-layer silica gel plate in a drying oven at 105 °C and activated it for 1–2 h before removing it. We used a pencil to draw a starting line 1 cm from the bottom of the silica gel plate and marked sample points every 1 cm. We used a capillary pipette to draw up the samples one by one and apply them to the sample points. After applying the samples, we dried them with a hair dryer. Pour the developing solvent into the chromatographic column and place the silica gel plate inside. When the developing solvent reaches 1 cm from the top of the plate, quickly remove the plate and dry it with a hairdryer. Use 10% sulfuric acid-ethanol as the developing agent, spray it evenly over the plate, and develop at 180 °C.

#### 2.5.2. Liquid Chromatography

Dissolve an appropriate amount of sample in methanol, sonicate until dissolved, filter through a 0.45 μm membrane, and set aside.

Chromatography column: ZORBAX Eclipse Plus C18 (Agilent Technologies Co., Ltd, Beijing, China)  (2.1 × 50 mm); column temperature: room temperature; mobile phase: 0–5 min (28% B), 5–15 min (28–32% B), 15–40 min (32–55% B), where mobile phase A is 0.5% phosphoric acid and B is acetonitrile; flow rate: 1.0 mL/min; detector: SPD-M20A diode array detector; wavelength: 205 nm; injection volume: 20 μL. 

#### 2.5.3. Infrared Spectroscopy

An amount of 1 mg of the sample was taken and mixed with Potassium Bromide (KBr), then ground to 1 mm sized particles for Fourier transform infrared spectroscopy (FT-IR) measurements. The infrared spectra (IR) of samples were obtained using an infrared spectrometer (Nicolet 6700, Thermo Fisher Nicolet, Waltham, MA, USA) in the frequency range of and detected in the frequency range of 700 to 4000 cm^−1^.

#### 2.5.4. Nuclear Magnetic Resonance Spectra

A total of 10 mg of the sample was taken and dissolved in 0.5 mL of deuterated methanol (CD_3_OD) at room temperature. ^1^H-NMR and ^13^C-NMR spectra were collected at 600 MHz and 151 MHz, respectively, on a Bruker AVANCE NEO spectrometer (Brucker, Karlsruhe, Germany).

#### 2.5.5. Mass Spectrometry

The chromatographic column refers to 2.5.2.

Mass spectrometry analysis was performed on the samples using the liquid-chromatography/mass spectrometry (LC-MS) system (Agilent 6546, Agilent Technologies Co., Ltd., Beijing, China). Mass spectrometry conditions: electrospray ionization source (ESI); capillary voltage: 3100 V, conical bore voltage: 15 V; reverse conical bore airflow: 25 L/h; ion source temperature: 100 °C, ion energy 1.0 V, induced dissociation energy: 5 V; positive ion scanning mode detection; detection range: 100–2000. The MS/MS in-source collision-induced dissociation (CID) voltage was 20–40 V.

### 2.6. Determination of S. nigrum Extract Antioxidant Activity

#### 2.6.1. DPPH Radical Scavenging Activity

The 2,2-diphenyl-1-picrylhydrazyl (DPPH) radical scavenging analysis was based on a previously described method [29] with slight modifications.

An amount of 1 mL of the sample solution (1, 2, 3, 4, and 5 mg/mL) was mixed with 1 mL of a 0.2 mmol/L ethanol solution containing 2,2-diphenyl-1-picrylhydrazyl (DPPH). The mixture was left to react in the dark at room temperature for 30 min. The absorbance value (A1) was measured at 517 nm. Subsequently, the DPPH ethanol solution was replaced with ultrapure water to determine the baseline absorbance (A2). The sample solution was then replaced with ultrapure water to measure the blank absorbance (A0). A vitamin C solution at the same concentration as the sample was used as a positive control. Three independent replicates were performed for each concentration, and average values were calculated. The calculation of the scavenging rate of DPPH radicals is shown in Equation (2). Half maximal inhibitory concentration (IC50) to the concentration of an antioxidant was required to reduce the concentration of free radicals (or other oxidants) by 50%. IC50 was calculated from dose–response curves using non-linear regression analysis. Sample concentrations were plotted against corresponding radical scavenging activity percentages, with IC50 values representing the concentration required to achieve 50% inhibition.

(2) $$\mathrm{DPPH}\;\mathrm{radical}\;\mathrm{scavenging}\;\mathrm{rate}\;(\%)=\left[1-\frac{A1-A2}{A0}\right]\times 100\%$$

where A1 is the absorbance of the sample group, A2 is the absorbance of the blank group, and A0 is the absorbance of the control group.

#### 2.6.2. Hydroxyl Radical Scavenging Activity

The hydroxyl radical scavenging analysis was based on a previously described method [30] with slight modifications. 

For each concentration, 0.5 mL of the sample solution was transferred to a reaction vessel. To this, 0.5 mL of 9.0 mmol/L ferrous sulfate solution and 0.5 mL of 9.0 mmol/L salicylic acid ethanol solution were added. The reaction was initiated by adding 0.25 mL of 8.8 mmol/L hydrogen peroxide solution. The solution was then reacted in a water bath at 37 °C for 30 min, followed by centrifugation at 679,333 × *g* for 5 min, the absorbance (A1) was measured at 510 nm. The sample background absorbance (A2) was determined by replacing the hydrogen peroxide with ultrapure water, while the blank absorbance (A0) was obtained by replacing the sample solution with ultrapure water. Vitamin C solution at equivalent concentrations was used as a positive control. All measurements were performed in triplicate, and average values were calculated. The calculation of the hydroxyl radical scavenging rate is shown in Equation (3).

(3) $$\mathrm{Hydroxyl}\;\mathrm{radical}\;\mathrm{scavenging}\;\mathrm{rate}\;(\%)=\left[1-\frac{A1-A2}{A0}\right]\times 100\%$$

where A1 is the absorbance of the sample group; A2 is the absorbance of the blank group; A0 is the absorbance of the control group. 

#### 2.6.3. ABTS Free Radical Scavenging Activity

The 2,2′-azino-bis(3-ethylbenzothiazoline-6-sulfonic acid) (ABTS) free radical scavenging analysis was based on a previously described method [31] with slight modifications.

An ABTS stock solution was prepared by mixing 7 mM 2,2′-azino-bis(3-ethylbenzothiazoline-6-sulfonic acid) (ABTS) and 2.45 mM potassium persulfate, followed by storage in the dark for 12 h. For each assay, a fresh ABTS working solution was prepared by diluting the stock solution with 10 mM phosphate buffer (pH 7.4) until an absorbance of 0.700 ± 0.02 at 734 nm was achieved. 

For analysis, 0.5 mL of sample solution (at varying concentrations) was combined with 1.5 mL of ABTS working solution. The mixture was incubated in the dark at room temperature for 6 min, after which its absorbance (A1) was measured at 734 nm. The sample background absorbance (A2) was determined by replacing the ABTS solution with ultrapure water, while the blank absorbance (A0) was obtained by replacing the sample solution with ultrapure water. Vitamin C solution at equivalent concentrations served as the positive control. All tests were conducted in triplicate, with mean values calculated. ABTS^+^ free radical scavenging rate is shown in Equation (4).

(4) $$\mathrm{ABTS}\;\mathrm{free}\;\mathrm{radical}\;\mathrm{scavenging}\;\mathrm{rate}\;(\%)=\left[1-\frac{A1-A2}{A0}\right]\times 100\%$$

where   A1  is the absorbance of the sample group, A2 is the absorbance of the blank group, and A0 is the absorbance of the control group. 

#### 2.6.4. Superoxide Anion Radical Scavenging Activity

Superoxide anion radical scavenging analysis based on a previously described method [32] with slight modifications.

For each concentration, 2 mL of sample solution was mixed with 4.5 mL of Tris(hydroxymethyl)aminomethane hydrochloride buffer solution (1 M, pH 8.2) and incubated at 37 °C for 30 min. Subsequently, 1 mL of 2 mM resorcinol was added to initiate the reaction. After 10 min of reaction, the absorbance (A1) was measured at 320 nm. The sample background absorbance (A2) was determined by replacing resorcinol with ultrapure water, while the blank absorbance (A0) was obtained by replacing the sample solution with ultrapure water. Vitamin C solution at equivalent concentrations served as the positive control. All measurements were performed in triplicate, with mean values calculated. The superoxide anion radical scavenging rate is shown in Equation (5).

(5) $$\mathrm{Superoxide}\;\mathrm{anion}\;\mathrm{radical}\;\mathrm{scavenging}\;\mathrm{rate}\;(\%)=\left[1-\frac{A1-A2}{A0}\right]\times 100\%$$

where A1 is the absorbance of the sample group, A2 is the absorbance of the blank group, and A0 is the absorbance of the control group.

#### 2.6.5. Ferric Ion Chelating Activity

Ferric ion reducing power analysis based on a previously described method [33] with slight modifications.

Sample solutions were prepared with concentration gradients of 1, 2, 3, 4, and 5 mg/mL. Take 0.5 mL of sample solution in a test tube and add 1 mL of phosphate buffer (0.2 mmol/L pH = 6.6). Mix well, then add 1 mL of 1% potassium ferricyanide solution. Place the tube in a 50 °C water bath for 30 min, then remove and allow it to cool down to room temperature. Next, add 1 mL of 10% trichloroacetic acid, mix well, and centrifuge at 169,833× *g* for 5 min. Aspirate 2.5 mL of the supernatant and add 2.5 mL of water and 0.5 mL of 0.1% ferric chloride. Mix well and react at 169,833× *g* for 5 min. Finally, measure the absorbance at 700 nm.

### 2.7. Measurement of Antimicrobial Activity of S. nigrum Extracts

The Oxford cup method [34,35] was used to determine the susceptibility of each sample to *Staphylococcus aureus* (*S. aureus*), *Escherichia coli* (*E. coli*), *Candida albicans* (*C. albicans*), *Shigella flexneri* (*S. flexneri*), *Pseudomonas aeruginosa* (*P. aeruginosa*), and *Salmonella paratyphi* B (*S. paratyphi* B).

Bacteria were filtered and removed using sterile 0.22 μm PTFE needle microporous membrane filters at concentrations of 1.25, 2.5, and 5 mg/mL for the five drug samples and the blank control, respectively. The bacterial suspensions were diluted to an Abs value of 0.8 ± 0.02 at 600 nm using nutrient broth (NB) medium and then set back. Each plate was divided equally into four areas and labeled. In total, 200 μL of bacterial broth at a concentration of 1 × 10^6^ CFU/mL was inoculated onto nutrient agar (NA) medium using the dilution smear plate method. An amount of 200 μL of 1.25, 2.5, and 5 mg/mL was added to the Oxford cups. The treated Petri dishes were sealed and incubated at 37 °C in a constant temperature incubator for 24 h, and the diameter of the zone of inhibition was measured.

### 2.8. Statistical Analysis

Experimental data are presented as “mean ± standard deviation (SD)” (n = 3) based on the results of three independent replicate experiments. Analysis of variance (ANOVA) used the Wallace–Duncan test. Organized data and calculations were performed using Excel 2021, and statistical analyses were conducted with SPSS Statistics 25.0, GraphPad, and Origin 2024. Statistical significance was defined at *p* < 0.05.

## 3. Results

### 3.1. Optimization of the Crude Saponin Extraction Process

As shown in Table S3, A2B2C2D1 was the optimal combination for the experiment, yielding the highest total saponin yield. In the extraction of saponins from *Solanum nigrum* fruit, the total saponin yield reached a maximum value of 8.59% at a temperature of 70 °C, continuous extraction for 4 h, ethanol concentration of 60%, and a material–liquid ratio of 1:20, which were the optimal extraction conditions.

According to the results of the range analysis, RA > RB > RC > RD, indicating that the order of importance is temperature > time > ethanol concentration > ratio of material to liquid. This suggested that, of the four factors, extraction temperature had the greatest impact on the saponin extraction rate of *Solanum nigrum* fruit.

### 3.2. Separation and Purification of Saponins for Identification of Results

#### 3.2.1. Thin-Layer Chromatography Analysis Results

The thin-layer chromatography results of the three samples purified from the separation were as follows.

As shown in Figure S1A, sample SNL1 was unfolded by chloroform–methanol (6:1), and the color was developed by 10% ethanol sulfate as pink spots with Rf = 0.2429. As shown in Figure S1B, sample SNL2 was unfolded with chloroform–methanol (6:0.2) and showed a pink spot after color development with Rf = 0.8919. As shown in Figure S1C, sample SNL3 was unfolded with chloroform–methanol (6:1.2), and the color developed into a purple-red spot with Rf = 0.1594. All the above samples had only one neat spot on the thin-layer chromatographic plate, which indicated that these three samples might be monomeric saponins.

#### 3.2.2. Liquid Chromatography Analysis Results

Area normalization calculations showed (Figure S2) that the purity of sample SNL1 was 89.42% with a retention time of 6.57 min, the purity of sample SNL2 was 91.89% with a retention time of 18.298 min, and the purity of sample SNL3 was 94.54% with a retention time of 5.526 min.

### 3.3. Structural Characterization Results of Saponins

#### 3.3.1. Structural Identification of the Saponin SNL1

**Figure 1:**
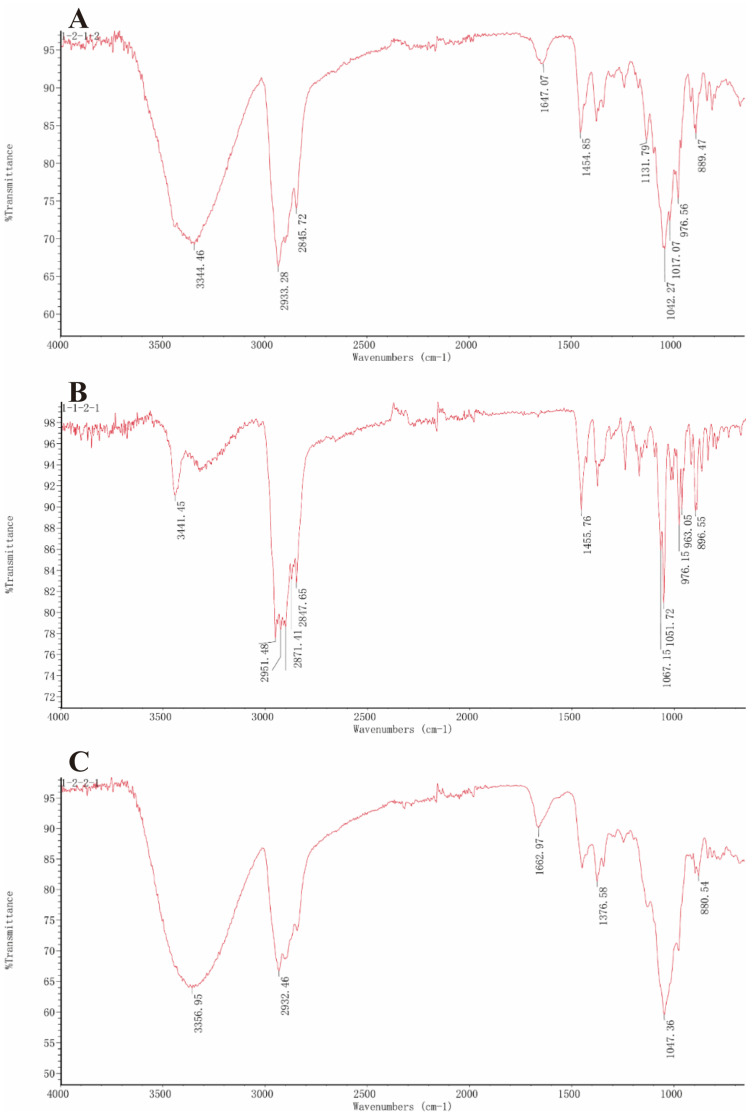
Infrared spectrogram of (**A**) SNL1, (**B**) SNL2, (**C**) SNL3. (The three monomers isolated and purified in this experiment were numbered SNL1, SNL2, and SNL3, respectively. The same abbreviations used subsequently refer to the same monomers.)

**Figure 2:**
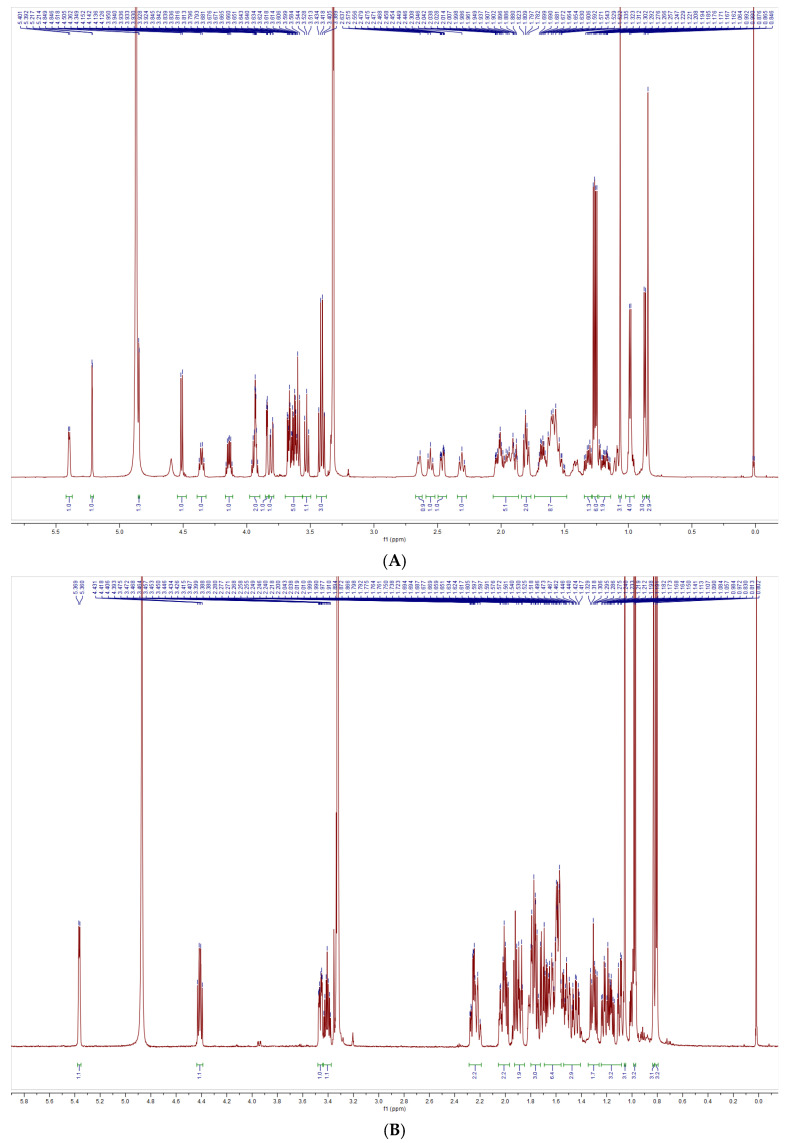

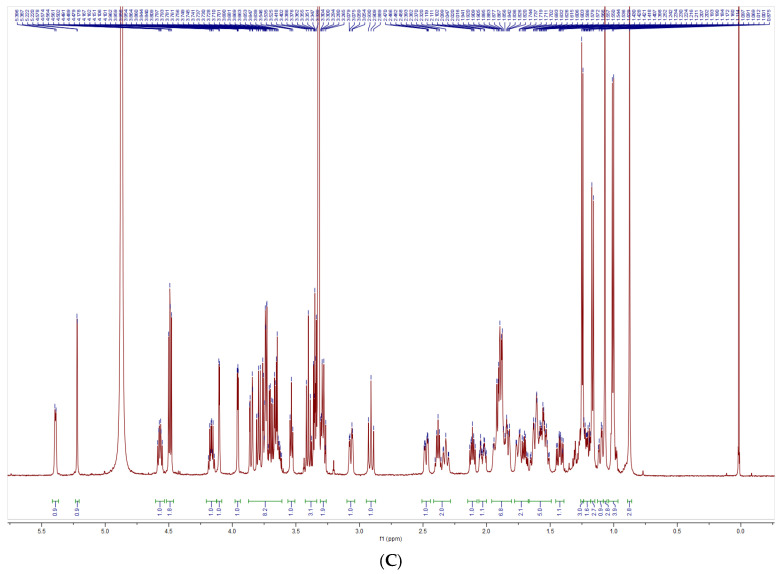
^1^H spectrometry of (**A**) SNL1, (**B**) SNL2, (**C**) SNL3.

**Figure 3:**
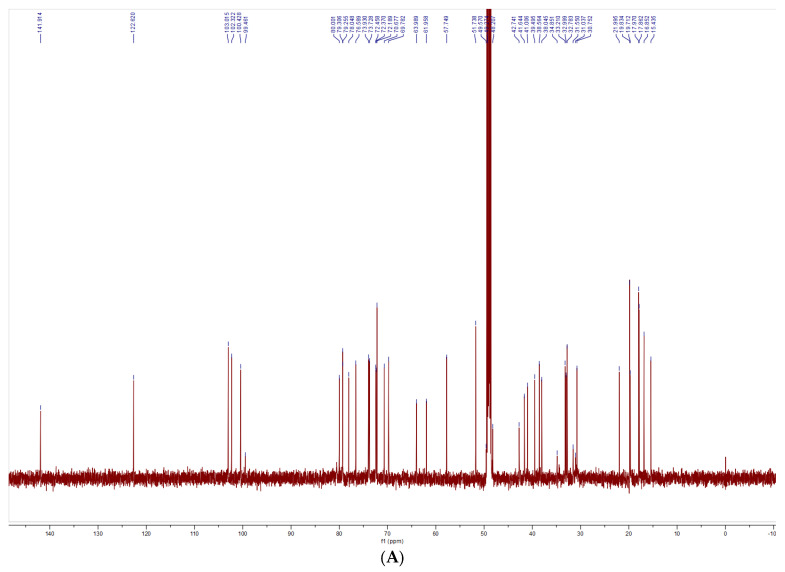

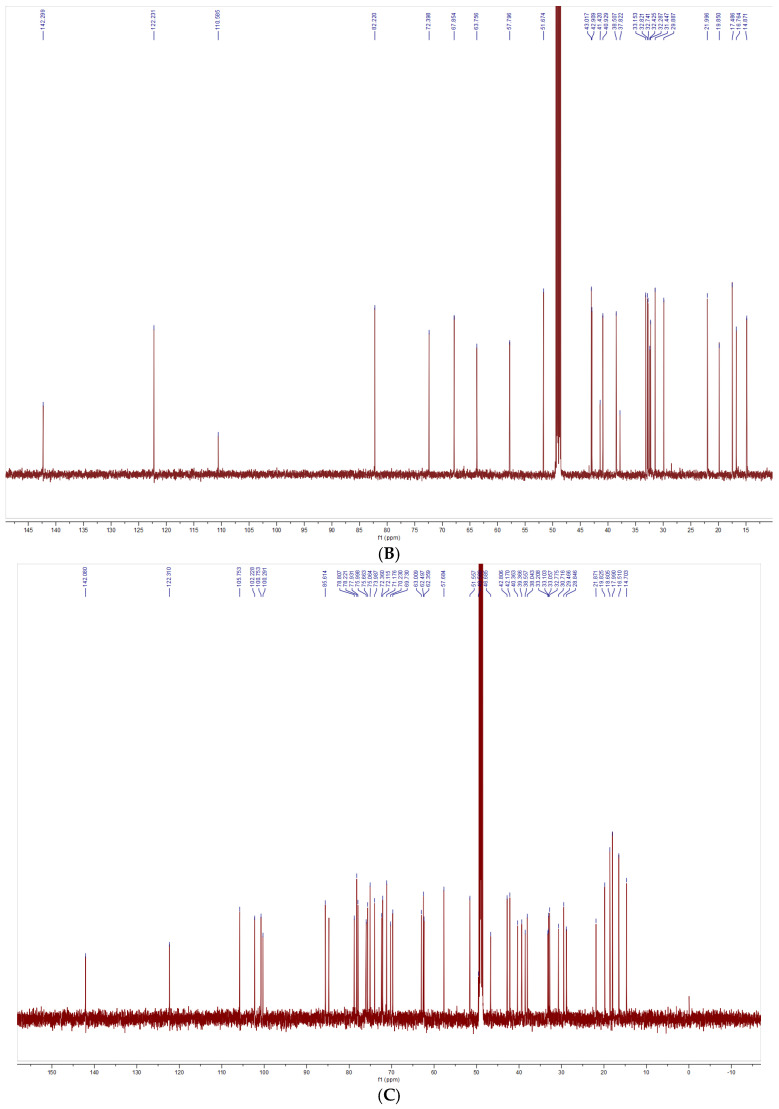
^13^C spectrometry of (**A**) SNL1, (**B**) SNL2, (**C**) SNL3.

**Figure 4:**
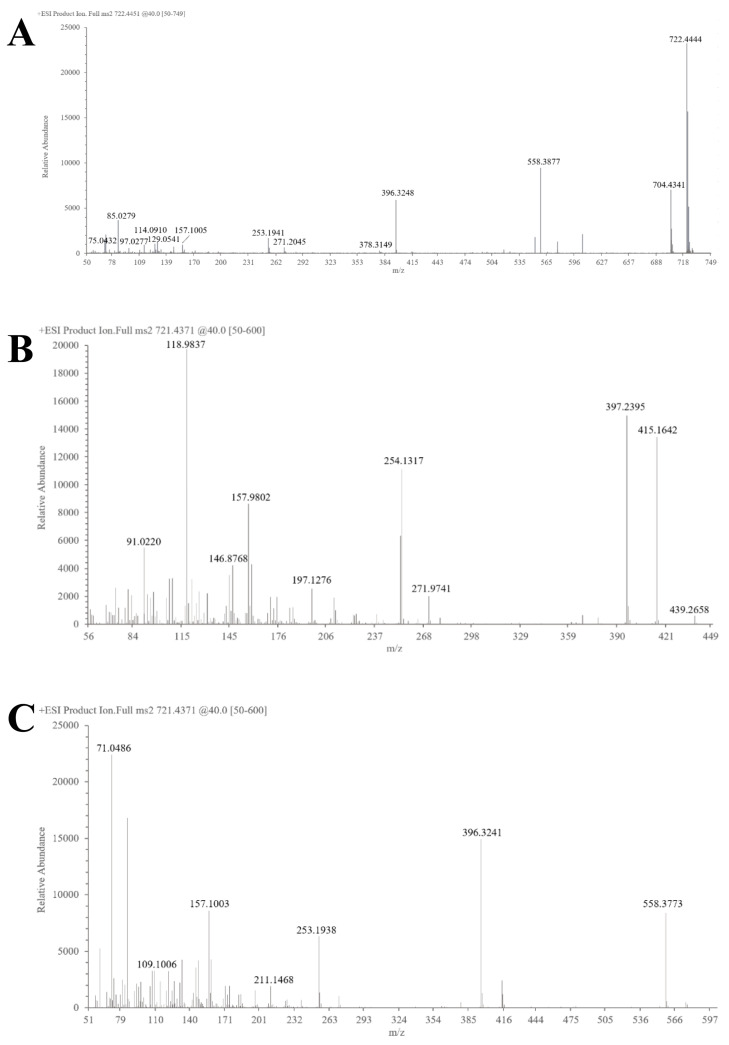
Mass spectrum of (**A**) SNL1, (**B**) SNL2, (**C**) SNL3.

**Figure 5:**
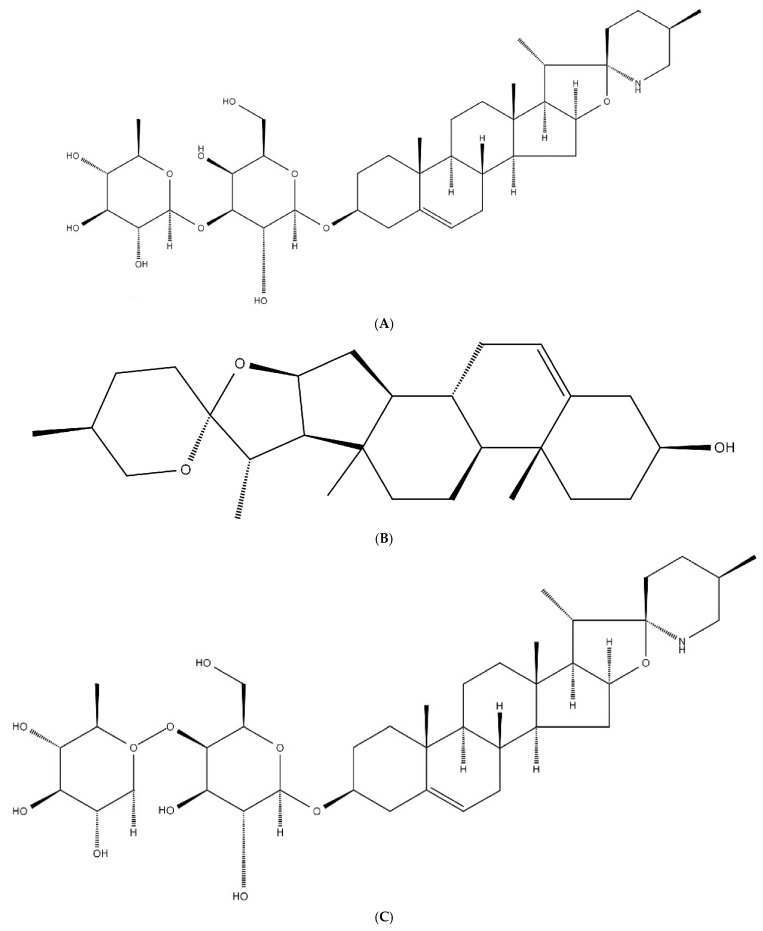
Structural formula of (**A**) SNL1, (**B**) SNL2, (**C**) SNL3.

The structural characterization of SNL1 was comprehensively elucidated through spectroscopic analyses. FT-IR spectra (Figure 1) displayed diagnostic absorption bands: a broad O-H stretching vibration at 3344 cm^−1^ indicative of hydroxyl-rich domains or hydrogen-bonding networks, aliphatic C-H stretches (2933–2846 cm^−1^) from sugar chain methyl groups, and critical markers including conjugated C=C (1647 cm^−1^), C-O-C asymmetric stretching (1132 cm^−1^), and a spiroketal signature at 977 cm^−1^. Complementary NMR analyses revealed key structural motifs: ^1^H NMR exhibited (Figure 2) olefinic protons (δ 5.21–5.40 ppm), broad hydroxyl signals (δ 1–6 ppm), and ether linkages (δ 3.39–3.96 ppm), while ^13^C NMR detected (Figure 3) 45 carbon signals with glycosidic C-O bonds (δ 41.01–80 ppm), consistent with hydrogen spectral data. ESI-MS ([M + H]^+^ 722) demonstrated (Figure 4) sequential fragmentation patterns (704 → 558 → 396 Da), revealing methyl hexose/pentose moieties through characteristic neutral losses (18, 146, 162 Da). Integrated spectral evidence confirmed SNL1 as γ2-solamargine (C_39_H_63_NO_11_), structurally characterized by a spirostan-type framework featuring hydroxylated cyclohexane units, a spiro-linked piperidine-oxapentacyclic system, and tri-substituted glycosylation (Figure 5). These findings align with established spectral databases [36], validating the compound’s identity through multi-technique correlation.

The molecular formula of γ2-Solamargine is C_39_H_63_NO_11_, and its chemical structure is shown in Figure 5. Its name is 2-[4,5-dihydroxy-2-(hydroxymethyl)-6-(5′,7,9,13-)tetramethylspiro[5-oxapentacyclo-18-en-6,2′-piperidin]-16-yl)oxacyclohexan-3-yl]oxy-6-methylcyclohexane-3,4,5-triol-Solamargine.

#### 3.3.2. Structural Identification of the Saponin SNL2

The structural elucidation of SNL2 was systematically validated through integrated spectroscopic analyses (Figure 1). FT-IR spectra revealed hydroxyl/hydrogen-bonding networks (3441 cm^−1^ O-H stretch), aliphatic C-H vibrations (2951 cm^−1^), and diagnostic spiroketal signatures (976/897 cm^−1^), complemented by conjugated C=C (1456 cm^−1^) and glycosidic C-O stretches (1067–1052 cm^−1^). NMR spectroscopy further resolved critical structural motifs: ^1^H NMR displayed olefinic protons (δ 5.36–5.37 ppm) and ether linkages (δ 3.38–3.48 ppm), while ^13^C NMR identified 27 carbon signals including conjugated alkenes (δ 122.23/142.30 ppm) and a characteristic spirostan C22 quaternary carbon (δ 110.59 ppm). ESI-MS ([M + H]^+^ 415) exhibited sequential fragmentation (397 → 272 → 158 Da), corresponding to neutral losses (18/125/114 Da) consistent with diosgenin derivatives. Multi-technique correlation confirmed SNL2 as diosgenin (C_27_H_42_O_3_), featuring a spirostan nucleus with a 5-oxapentacyclic system and a 16-hydroxyl group [36,37,38]. 

Taken together, the secondary saponin SNL2 was consistent with diosgenin, which was determined to have a molecular formula of C_27_H_42_O_3_ and a chemical structure as shown in Figure 5 , with the name S,2S,4S,5′R,6R,7S,8R,9S,12S,13R,16S)-5′,7,9,13-tetramethylspiro[5-oxapentacyclo[10.8.0.02,9.04,8.013,18]icos-18-ene-6,2′-oxane]-16-ol.      

#### 3.3.3. Structural Identification of the Saponin SNL3

The structural characterization of SNL3 was confirmed through integrated spectral analyses (Figure 1). FT-IR revealed hydroxyl-rich domains (3357 cm^−1^ O-H stretch), aliphatic C-H vibrations (2932 cm^−1^), conjugated C=C (1663 cm^−1^), and diagnostic spiroketal absorption (881 cm^−1^). NMR spectroscopy identified critical motifs: ^1^H NMR displayed olefinic protons (δ 5.22–5.40 ppm), hydroxyl clusters (δ 1–6 ppm), and ether linkages (δ 3.27–3.96 ppm), while ^13^C NMR resolved 45 signals including alkene carbons (δ 122.31/142.08 ppm), glycosidic C-O bonds (δ 40.36–78.81 ppm), and C-N bonds (δ 30.72–63.01 ppm). ESI-MS ([M + H]^+^ 721) exhibited sequential hexose losses (162 Da × 2), yielding fragments at m/z 559 and 397. Multi-technique correlation established SNL3 as β-solanine (C_39_H_63_NO_11_), featuring a steroidal spiroketal nucleus with bis-glycosylation at C_3_ and C_27_ positions (Figure 5), consistent with literature spectral databases [39].

Based on the above analysis, the secondary saponin SNL3 was consistent with beta-solanine, and its molecular formula is determined to be C_39_H_63_NO_11_. The chemical formula structure is shown in Figure 5, and its name is (2*S*,3*R*,4*S*,5*S*,6*R*)-2-[(2*R*,3*S*,4*S*,5*R*,6*R*)-3,5-dihydroxy-2-(hydroxymethyl)-6-[[(1*S*,2*S*,7*S*,10*R*,11*S*,14*S*,15*R*,16*S*,17*R*,20*S*,23*S*)-10,14,16,20-tetramethyl-22-azahexacyclo[12.10.0.0^2,11^.0^5,10^.0^15,23^.0^17,22^]tetracos-4-en-7-yl]oxy]oxan-4-yl]oxy-6-(hydroxymethyl)oxane-3,4,5-triol.

#### 3.4. In Vitro Antioxidant Activity Analysis of Extracts

##### 3.4.1. DPPH Free Radical Scavenging Capacity

**Figure 6:**
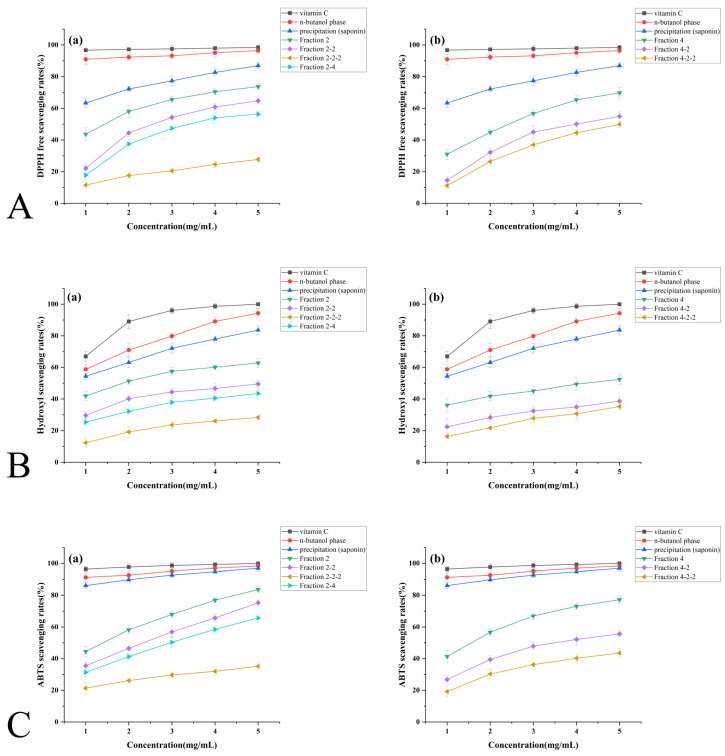

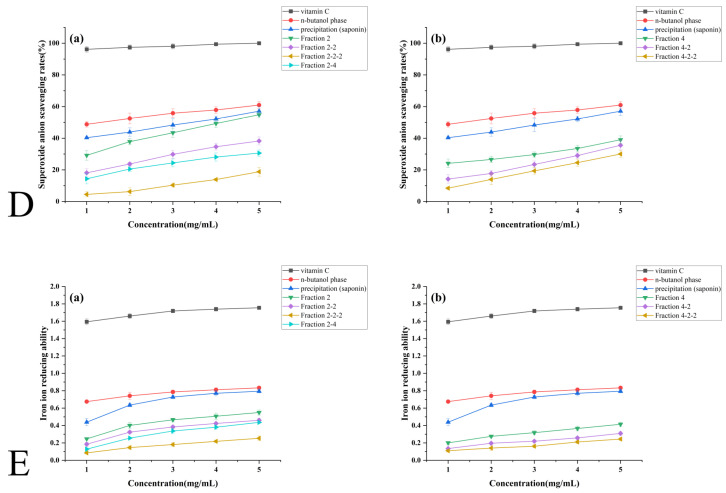
Determination of antioxidant activity of saponins during purification of *Solanum nigrum* fruits. (**A**) DPPH free radical scavenging capacity. (**B**) Hydroxyl radical scavenging capacity. (**C**) ABTS free radical scavenging capacity. (**D**) Superoxide anion radical scavenging capacity. (**E**) Ferric ion reducing power capacity.

Figure 6A illustrates the DPPH radical scavenging activity of *Solanum nigrum* saponins during purification. All fractions exhibited concentration-dependent responses (1–5 mg/mL), with the *n*-butanol extract achieving 97.99% scavenging at 5 mg/mL—comparable to vitamin C. Precipitated saponins demonstrated linear scavenging correlation (86.94% at 5 mg/mL).

Purified fractions 2, 2-2, and 2-2-2 (SNL1) showed progressive scavenging enhancement up to 4 mg/mL before plateauing, while fraction 2-4 (SNL2) maintained linear dose-dependence throughout 1–5 mg/mL. Group (b) fractions 4, 4-2, and 4-2-2 (SNL3) displayed scavenging efficiencies of 69.81%, 54.90%, and 49.85%, respectively, at 5 mg/mL.

After curve fitting calculations, the IC50 valued for each sample are as follows. The lower the IC50 value, the stronger the antioxidant activity:

Group (a): vitamin C (0.00018 mg/mL) > *n*-butanol phase (0.0096 mg/mL) > saponin precipitate (0.51 mg/mL) > Fraction 2 (1.36 mg/mL) > Fraction 2-2 (2.69 mg/mL) > Fraction 2-2-2 (3.54 mg/mL) > Fraction 2-4 (21.94 mg/mL).

Group (b): vitamin C (0.00018 mg/mL) > *n*-butanol phase (0.0096 mg/mL) > saponin precipitate (0.51 mg/mL) > Fraction 4 (2.27 mg/mL) > Fraction 4-2 (3.94 mg/mL) > Fraction 4-2-2 (4.83 mg/mL).

##### 3.4.2. Hydroxyl Radical Scavenging Capacity

Figure 6B demonstrates the hydroxyl radical scavenging activity of *Solanum nigrum* saponins during purification. Both *n*-butanol extracts and saponin precipitates exhibited linear concentration-dependent responses (1–5 mg/mL), achieving 94.28% and 83.63% scavenging at 5 mg/mL, respectively.

Purified fractions (2, 2-2, 2-2-2, 2-4) showed progressive scavenging enhancement up to 3 mg/mL, followed by reduced efficacy gains at higher concentrations. In purified fractions (4, 4-2, 4-2-2), scavenging efficiency increased minimally with concentration, reaching 38.68% and 35.28% at 5 mg/mL for 4-2 and 4-2-2, respectively.

IC50 values ranked as follows:

Group (a): vitamin C (0.73 mg/mL) > *n*-butanol phase (0.8 mg/mL) > saponin precipitate (0.89 mg/mL) > Fraction 2 (1.82 mg/mL) > Fraction 2-2 (4.97 mg/mL) > Fraction 2-2-2 (8.31 mg/mL) > Fraction 2-4 (21.49 mg/mL).

Group (b): vitamin C (0.73 mg/mL) > *n*-butanol phase (0.8 mg/mL) > saponin precipitate (0.89 mg/mL) > Fraction 4 (4.32 mg/mL) > Fraction 4-2 (13.44 mg/mL) > Fraction 4-2-2 (13.94 mg/mL).

##### 3.4.3. ABTS Free Radical Scavenging Capacity

Figure 6C demonstrates ABTS radical scavenging activity during *Solanum nigrum* saponin purification. The *n*-butanol phase and saponin precipitates achieved near-vitamin C efficacy at 5 mg/mL (98.40% and 95.94%, respectively), indicating superior ABTS targeting compared to other radicals.

Purified fractions (2, 2-2, 2-2-2) exhibited dose-dependent scavenging (65.69–83.61% at 5 mg/mL), while fractions 2-4 plateaued at 35.23% efficacy. Fractions (4, 4-2, 4-2-2) showed diminishing returns with concentration escalation, culminating in 38.68% and 35.28% scavenging for 4-2 and 4-2-2 at 5 mg/mL.

The IC50 values for each sample are as follows:

Group (a): vitamin C (0.055 mg/mL) > *n*-butanol (0.061 mg/mL) > saponin precipitate (0.104 mg/mL) > Fraction 2 (1.33 mg/mL) > Fraction 2-2 (2.05 mg/mL) > Fraction 2-2-2 (2.707 mg/mL) > Fraction 2-4 (22.2 mg/mL).

Group (b): vitamin C (0.055 mg/mL) > *n*-butanol (0.061 mg/mL) > saponin precipitate (0.104 mg/mL) > Fraction 4 (1.46 mg/mL) > Fraction 4-2 (3.558 mg/mL) > Fraction 4-2-2 (6.923 mg/mL).

##### 3.4.4. Superoxide Anion Radical Scavenging Capacity

Figure 6D demonstrates superoxide anion radical scavenging during *Solanum nigrum* saponin purification. Both *n*-butanol extracts and saponin precipitates showed limited concentration responsiveness (60.96% and 57.09% scavenging at 5 mg/mL, respectively), while purified Fraction 2 achieved comparable efficacy (55.85%) through progressive enhancement.

Dose-dependent responses were observed in Fractions 2-2 and 2-2-2, whereas Fraction 2-4 exhibited accelerated scavenging above 2 mg/mL. After curve fitting calculations, the IC50 values for each sample are as follows. IC50 values revealed consistent hierarchies:

Group (a): vitamin C (0.045 mg/mL) > *n*-butanol (1.29) > saponin precipitate (3.077) > 2 (4.08) > 2-2 (10.51) > 2-2-2 (17.51) > 2-4 (19.43).

Group (b): vitamin C (0.045) > *n*-butanol (1.29) > saponin (3.077) > 4-2 (11.36) > 4-2-2 (12.00) > 4 (17.79).

Notably, Fractions 4 (39.05%), 4-2 (35.56%), and 4-2-2 (30.03%) at 5 mg/mL displayed parallel concentration–response patterns despite weak overall efficacy, confirming conserved structure–activity relationships across purification stages.

##### 3.4.5. Ferric Ion Reducing Power Capacity

Figure 6E illustrates superoxide anion radical scavenging via ferric ion reduction capacity during Solanum saponin purification.

The *n*-butanol phase exhibited concentration-independent ferric reduction (plateaued at 5 mg/mL), while saponin precipitates showed marked enhancement from 1 to 3 mg/mL before stabilization. Purified Group (a) fractions (2, 2-2, 2-2-2) demonstrated dose-dependent responses, with 2-2-2 achieving comparable efficacy to 2-2 at 5 mg/mL. In contrast, fractions 2-4 displayed negligible concentration responsiveness (35.23% at 5 mg/mL).

Purified Group (b) fractions (4, 4-2, 4-2-2) maintained weak reducing capacities with parallel concentration–response curves, confirming consistency between redox activity and antioxidant performance.

### 3.5. Antimicrobial Activity of S. nigrum Extracts

#### 3.5.1. Bacteriostatic Effect on *Escherichia coli*

Table 1 demonstrates the concentration-dependent antibacterial effects of *Solanum nigrum* fruit saponin components against *Escherichia coli*. All tested fractions exhibited dose-responsive inhibition, with superior activity observed in the n-butyl alcohol phase compared to saponin precipitates at 1.25–2.5 mg/mL. This trend reversed at 5 mg/mL, where saponin precipitates showed enhanced efficacy.

Purified Fraction 2 displayed exceptional antibacterial performance, achieving a 17.25 mm inhibition zone at 5 mg/mL after demonstrating sharp activity escalation from 2.5 mg/mL. However, subsequent purification generally reduced antimicrobial potency—fractions 2-4 and 2-2 showed diminished activity compared to parent Fraction 2, with further attenuation observed in subfractions 2-2-2. Similar activity reduction patterns occurred in the 4-series fractions across the 1.25–5 mg/mL range. Intermediate concentrations (2.5–5 mg/mL) maintained relatively stable antibacterial trends, though purified derivatives consistently underperformed compared to their crude counterparts.

#### 3.5.2. Bacteriostatic Effect on *Candida albicans*

Table 2 reveal concentration-dependent antifungal activity of *Solanum nigrum* saponin fractions against *Candida albicans*, with *n*-butanol phase and fractions 2-4/4-2-2 showing no inhibition. Component 2 demonstrated optimal efficacy (17 mm inhibition zone at 5 mg/mL), while purified components 2 and 4 exhibited superior activity to crude saponin precipitates.

Progressive purification generally reduced antifungal potency: successive activity reduction occurred in fraction series 2 (2 > 2-2 > 2-2-2) and 4 (4 > 4-2 > 4-2-2), which was in line with the trend of the inhibitory effect on *E. coli.*

#### 3.5.3. Bacteriostatic Effect on *Shigella flexneri*

The inhibition of *Shigella flexneri* by each component of the saponins during *Solanum nigrum* fruit purification is shown in Table 3.

As can be seen from the table, most of the components showed an improved inhibitory effect on *Shigella flexneri* with increasing concentration. In the concentration range of 1.25–2.5 mg/mL, the antibacterial effect of the precipitate (saponins) was better than that of the *n*-butanol phase. After primary purification, component 2 had the best antibacterial effect on Shigella, and the antibacterial zone reached 17 mm at 5 mg/mL, indicating an excellent antibacterial effect. In comparison, the antibacterial effect of components 2-2 and 2-4 purified from component 2 was significantly weaker than that of unpurified component 2. Further purification of component 2-2-2 showed no bacteriostatic effect. Similarly, in the concentration range of 1.25–5 mg/mL, the bacteriostatic ability of component 4 purified from precipitate (saponins) was slightly lower than that of crude saponins. The antibacterial zone of fractions 4-2 and 4-2-2 purified from fraction 4 was not detected, which was consistent with the above trend.

#### 3.5.4. Bacteriostatic Effect on *Salmonella paratyphi* B

The inhibition of *Salmonella paratyphi* B by each component of the saponins during *Solanum nigrum* fruit purification is shown in Table 4.

As can be seen from the table, the inhibitory effect of each component on *S. paratyphi* B was weaker than that of the above strains. Only precipitant (saponin), fraction 2, fraction 2-2, and fraction 4 had a concentration-dependent inhibitory effect on Salmonella, and no inhibitory zone was detected in the other groups. The bacteriostatic effect of fraction 2 was slightly better than that of precipitate (saponin), and that of fraction 4 was slightly worse than that of precipitate (saponin). The bacteriostatic ability of fraction 2-2 purified from fraction 2 decreased, which was consistent with the bacteriostatic trend of the above bacteria.

#### 3.5.5. Bacteriostatic Effect on *Pseudomonas aeruginosa*

The inhibition of *Pseudomonas aeruginosa* by each component of the saponins during *Solanum nigrum* fruit purification is shown in Table 5.

The precipitate (saponins) showed a slight inhibitory effect on *P. aeruginosa*, and the inhibition zone was 13 mm at 5 mg/mL. No inhibition zone was detected in other fractions, indicating that there was no inhibitory effect on *P. aeruginosa*.

#### 3.5.6. Bacteriostatic Effect on *Staphylococcus aureus*

During the purification of *Solanum nigrum* fruits, no inhibition zone against *Staphylococcus aureus* was detected for each component of the saponins, indicating that there was no inhibitory effect on *Staphylococcus aureus.*

## 4. Discussion

The antioxidant capacity of *Solanum nigrum* saponins shows a marked inverse correlation with purification intensity, with semi-purified fractions exhibiting enhanced radical scavenging and reducing power compared to highly purified monomers [40]. This functional decrease is likely due to synergistic phytochemical depletion and structural modifications during purification. Moderately purified fractions retain co-extractives, such as phenolic-polysaccharide matrices, which facilitate electron donation and Fe^2+^ chelation, thereby optimizing reactive oxygen species (ROS) neutralization. Meanwhile, the antioxidant activity of Fraction 2-2-2 and 4-2-2 is significantly stronger than that of Fraction 2-4. This may be because the former two have relatively longer, chain-like structures containing a relatively large number of active hydroxyl groups, as well as side chains attached at the 3-position. These side chains may serve as potential structural domains for antioxidant activity, enabling them to participate more effectively in antioxidant reactions. Exhaustive purification isolates truncated monomers (e.g., γ^2^-solamargine [SNL1] and β-solanine [SNL3]), characterized by shortened saccharide chains and reduced hydroxyl and ether functionalities, which impair ROS stabilization. Deglycosylated sapogenins also aggregate hydrophobically via steroidal core interactions, hindering electron transfer kinetics. The remarkably weak superoxide anion (O_2_) scavenging of purified saponins reflects molecular inadequacy—short-chain derivatives lack structural complementarity to O_2_ transition states—and silica gel chromatography systematically excludes synergistic complexes essential for sustained antioxidant activity. Crucially, hydrogen bonding networks within semi-purified systems stabilize saponin conformations and enhance functionality. These findings highlight the limitations of purity-centered approaches and argue for functionally optimized partial purification to preserve phytocomplex integrity and maximize antioxidant efficacy. This positions semi-purified fruit extracts as optimal active ingredients for food preservation.

Parallel observations emerge in antibacterial assessments, where semi-purified fractions surpass both crude extracts and purified monomers in efficacy—a phenomenon similarly rooted in synergy erosion and structural incompatibility. Partial purification preserves co-solubilizing agents that enhance saponin–membrane interactions, while exhaustive isolation yields truncated-chain monomers (SNL1/SNL3) incapable of penetrating Gram-negative outer membranes [41]. Hydrophobically aggregated sapogenins (SNL2) further fail to disrupt lipid bilayers. Species-specific barriers modulate outcomes: the thick peptidoglycan layer characteristic of Gram-positive bacteria physically excludes saponin access, while *Pseudomonas aeruginosa* necessitates multi-target inhibition—a capability eradicated during purification due to loss of effector diversity [42]. Silica gel chromatography’s polarity bias exacerbates this limitation, discarding polar co-factors critical for broad-spectrum activity.

Future development should prioritize hybrid saponin derivatives with extended sugar chains and co-extractive blends, positioning *S. nigrum* saponins as sustainable dual-functional agents for food preservation and antimicrobial applications. We can take the semi-purified extract derived from fruits (non-isolated saponins) as the main active ingredient. Microencapsulation of fruit-based extracts holds particular promise for scalable implementation in the food industry, as it balances efficacy with stability where purified monomers often fail.

## 5. Conclusions

This study establishes *Solanum nigrum* fruit as a viable source of saponins with dual functionality. Through optimized ethanol extraction and sequential purification, three saponins were structurally characterized as γ2-solamargine (SNL1), diosgenin (SNL2), and β-solanine (SNL3) by IR, NMR, and MS analyses. We demonstrated that the crude n-butanol fraction, enriched with saponin–polyphenol–polysaccharide complexes, outperformed purified monomers in both antioxidant and antimicrobial activities. This synergy, attributed to non-covalent interactions such as hydrogen bonding or hydrophobic associations, highlights the practical advantage of minimally processed plant extracts. Such extracts not only retain bioactive complexity but also circumvent energy-intensive purification steps, aligning with circular economy principles by valorizing agricultural byproducts into low-cost, eco-friendly preservatives. The superior performance of crude fractions suggests a shift toward leveraging natural phytochemical synergies rather than isolated compounds. Natural product purification requires a ‘moderate purification’ strategy that retains key cofactors rather than pursuing absolute purity, and consideration should be given to the development of complex antimicrobials based on the synergistic interaction of components. However, challenges remain in stabilizing these natural complexes against environmental stressors and standardizing their bioactivity for industrial scalability. Future research should prioritize encapsulation technologies to enhance stability and employ response surface methodology to optimize synergistic ratios for targeted food matrices, such as dairy or meat products. Our findings advance the utilization of under-exploited medicinal edible plants in sustainable food preservation strategies.

## Figures and Tables

**Table 1:** Bacteriostatic effect on *Escherichia coli* (Antibacterial circle diameter: mm).

	1.25 mg/mL	2.5 mg/mL	5 mg/mL
*n*-butanol phase	14.00 ± 1.00 **	14.50 ± 1.50 **	16.13 ± 2.37 **
Precipitation (saponin)	13.00 ± 0.50 ***	14.25 ± 1.00 ***	16.75 ± 1.00 **
Fr. 2	12.25 ± 1.50 **	17.00 ± 0.75 ***	17.25 ± 0.75 **
Fr. 2-2	13.25 ± 0.50 ***	14.50 ± 0.50 ***	14.75 ± 1.50 **
Fr. 2-2-2	12.75 ± 0.25 ***	14.25 ± 0.75 ***	14.25 ± 1.00 ***
Fr. 2-4	11.17 ± 0.83 **	11.67 ± 1.33 **	12.00 ± 0.75 ***
Fr. 4	13.17 ± 0.33 ***	13.75 ± 0.75 ***	14.00 ± 1.00 ***
Fr. 4-2	12.75 ± 0.75 ***	13.50 ± 0.50 ***	13.75 ± 0.75 ***
Fr. 4-2-2	12.25 ± 0.25 ***	12.33 ± 0.17 ***	12.50 ± 0.50 ***

Note: “**” indicates *p* ≤ 0.01; “***” indicates *p* ≤ 0.001.

**Table 2:** Antibacterial effect on *Candida albicans* (antibacterial circle diameter: mm).

	1.25 mg/mL	2.5 mg/mL	5 mg/mL
*n*-butanol phase	—	—	—
Precipitation (saponin)	—	14.00 ± 1.25 **	16.25 ± 1.00 ***
Fr. 2	14.00 ± 0.75 ***	15.00 ± 1.75 **	17.00 ± 1.75 **
Fr. 2-2	12.50 ± 0.50 ***	13.50 ± 0.75 ***	15.75 ± 1.00 **
Fr. 2-2-2	—	—	10.50 ± 0.50 ***
Fr. 2-4	—	—	—
Fr. 4	13.25 ± 0.25 ***	15.5 ± 0.50 ***	16.75 ± 0.75 ***
Fr. 4-2	10.50 ± 0.50 **	13.00 ± 0.75 ***	14.00 ± 1.00 **
Fr. 4-2-2	—	—	—

Note: “**” indicates *p* ≤ 0.01; “***” indicates *p* ≤ 0.001; “—” indicates no bacteriostatic effect.

**Table 3:** Bacteriostatic effect on *Shigella flexneri* (Antibacterial circle diameter: mm).

	1.25 mg/mL	2.5 mg/mL	5 mg/mL
*n*-butanol phase	12.50 ± 0.50 ***	14.50 ± 0.50 ***	15.50 ± 1.00 **
Precipitation (saponin)	13.83 ± 1.66 **	15.50 ± 0.75 ***	15.83 ± 1.66 **
Fr. 2	15.25 ± 0.25 ***	16.00 ± 0.75 ***	17.00 ± 1.00 ***
Fr. 2-2	10.50 ± 0.75 **	14.67 ± 0.83 **	15.75 ± 0.75 ***
Fr. 2-2-2	—	—	—
Fr. 2-4	12.50 ± 0.25 ***	12.50 ± 0.50 ***	12.67 ± 0.33 ***
Fr. 4	13.00 ± 0.75 ***	15.25 ± 0.75 ***	15.50 ± 0.50 ***
Fr. 4-2	—	—	—
Fr. 4-2-2	—	—	—

Note: “**” indicates *p* ≤ 0.01; “***” indicates *p* ≤ 0.001; “—” indicates no bacteriostatic effect.

**Table 4:** Bacteriostatic effect on *Salmonella* (Antibacterial circle diameter: mm).

	1.25 mg/mL	2.5 mg/mL	5 mg/mL
*n*-butanol phase	—	—	—
Precipitation (saponin)	11.00 ± 1.00 **	12.25 ± 0.75 ***	12.67 ± 0.83 ***
Fr. 2	11.17 ± 1.33 *	12.33 ± 0.67 ***	12.75 ± 0.75 ***
Fr. 2-2	10.00 ± 0.25 ***	10.33 ± 0.17 ***	10.50 ± 0.25 ***
Fr. 2-2-2	—	—	—
Fr. 2-4	—	—	—
Fr. 4	10.00 ± 0.25 ***	11.17 ± 0.33 ***	11.67 ± 0.83 **
Fr. 4-2	—	—	—
Fr. 4-2-2	—	—	—

Note: “*” indicates *p* ≤ 0.05;“**” indicates *p* ≤ 0.01; “***” indicates *p* ≤ 0.001; “—” indicates no bacteriostatic effect.

**Table 5:** Bacteriostatic effect on *Pseudomonas aeruginosa* (antibacterial circle diameter: mm).

	1.25 mg/mL	2.5 mg/mL	5 mg/mL
*n*-butanol phase	—	—	—
Precipitation (saponin)	11.50 ± 0.25 ***	12.50 ± 1.00 **	13.00 ± 0.50 ***
Fr.2	—	—	—
Fr.2-2	—	—	—
Fr.2-2-2	—	—	—
Fr.2-4	—	—	—
Fr.4	—	—	—
Fr.4-2	—	—	—
Fr.4-2-2	—	—	—

Note: “**” indicates *p* ≤ 0.01; “***” indicates *p* ≤ 0.001; “—” indicates no bacteriostatic effect.

## Data Availability

The original contributions presented in the study are included in the article/Supplementary Materials, further inquiries can be directed to the corresponding author.

## Supplementary Materials

The following supporting information can be downloaded at https://www.mdpi.com/article/10.3390/foods14132370/s1, Figure S1:Thin-layer chromatography results of (A) SNL1, (B) SNL2, and (C) SNL3.(Numbered zones represent experimental reference codes for TLC spot tracking (no quantitative significance). The three monomers isolated and purified in this experiment were numbered SNL1, SNL2, and SNL3, respectively. The same abbreviations used subsequently refer to the same monomers.); Figure S2. Liquid chromatographic results of (A) SNL1, (B) SNL2, and (C) SNL3.; Table S1: Factors and levels of the single-factor experiments(Solid-liquid ratio refers to the proportional relationship between the weight of dry plant material (g) and volume of extraction solvent (mL) in extraction.); Table S2. Orthogonal experiment on the extraction technology of *Solanum nigrum.; *Table S3. Results of the orthogonal test on the extraction conditions of *Solanum nigrum* fruit.
