# Supplementary material for: Saponins from Solanum nigrum L. Fruit: Extraction Optimization, Structural Characterization, and Dual-Functional Efficacy
Source: Foods. 2025 Jul 3;14(13):2370. doi: 10.3390/foods14132370 (PMC12249067; doi:10.3390/foods14132370)
Supplement: Supplementary file 1 [file foods-14-02370-s001.zip › foods-3670954-supplementary.pdf]

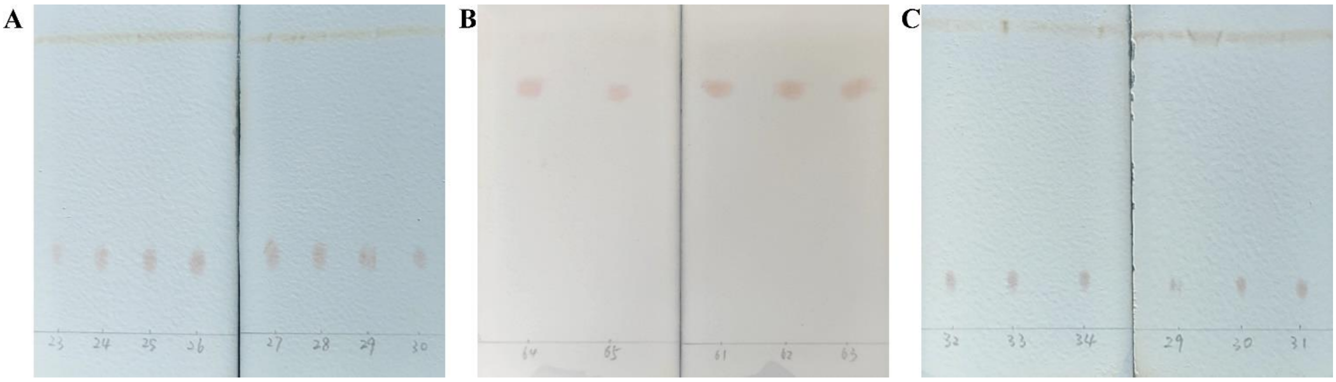

Figure S1. Thin-layer chromatography results of (A)SNL1,(B)SNL2, and (C)SNL3.(Numbered zones represent experimental reference codes for TLC spot tracking (no quantitative significance). The three monomers isolated and purified in this experiment were numbered SNL1, SNL2, and SNL3, respectively. The same abbreviations used subsequently refer to the same monomers.)

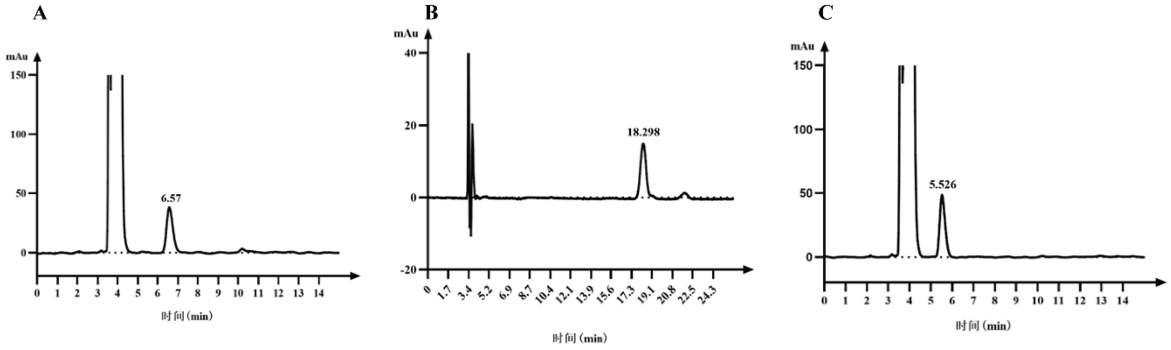

Figure S2. Liquid chromatographic results of (A)SNL1,(B)SNL2, and (C)SNL3.

Table S1. Factors and levels of the single-factor experiments(Solid-liquid ratio refers to the proportional relationship between the weight of dry plant material (g) and volume of extraction solvent (mL) in extraction.)

**Independent variables**	**Levels**
Extraction temperature	40℃,50℃,60℃,70℃,80℃
Extraction time	1h, 2h, 3h, 4h, 5h
Solvent concentration	40%、50%、60%、70%、80%、90%
Solid-Liquid Ratio	1:10、1:15、1:20、1:25、1:30

Table S2. Orthogonal experiment on the extraction technology of *Solanum nigrum*.

**Levels**	**Factors**			
	Extraction temperature（℃）	Extraction time（h）	Solvent concentration（%）	Solid-Liquid Ratio
1	60	3	50	1:20
2	70	4	60	1:25
3	80	5	70	1:30

Table S3. Results of the orthogonal test on the extraction conditions of *Solanum nigrum* fruit

Trial No.	A Temperature (°C)	B Time (h)	C Ethanol Concentration (%)	D Solid-Liquid Ratio	Total Saponin Yield (%)
1	1	1	1	1	7.34
2	1	2	3	2	7.65
3	1	3	2	3	8.14
4	2	1	3	3	7.96
5	2	2	2	1	8.59
6	2	3	1	2	8.27
7	3	1	2	2	7.95
8	3	2	1	3	8.16
9	3	3	3	1	8.36
K1	23.13	23.25	23.77	24.29	
K2	24.82	24.40	24.68	23.87	
K3	24.47	24.77	23.97	24.26	
k1	7.71	7.75	7.92	8.10	
k2	8.27	8.13	8.23	7.96	
k3	8.16	8.26	7.99	8.09	
R	0.56	0.51	0.30	0.14	
Primary-secondary order	A > B > C > D				
Optimal Level	A2	B3	C2	D1	
Optimal Combination	A2B2C2D1				
